# Discrete papular lichen myxedematosus: a rare entity or an under- diagnosed disease?

**DOI:** 10.11604/pamj.2014.19.180.5389

**Published:** 2014-10-21

**Authors:** Iman Hadj, Salim Gallouj, Mariame Meziane, Fatima Zahra Mernissi

**Affiliations:** 1 Department of Dermatology, CHU Hassan II, Fez, Morocco

**Keywords:** Mucinosis-discrete, papular lichen, myxedematosus

## Abstract

Primary cutaneous mucinoses are characterized by abnormal mucin deposits in the skin. Discrete papular lichenmyxedematosus (DPLM) is an unusual subtype which is characterized by the presence of multiples smooth, waxy, or flesh-colored papules, 2 to 5 mm in size affecting the trunk and limbs and most commonly proximal sites. We report a 42-year-old man with both the clinical and histopathological described criteria.

## Introduction

Cutaneous mucinoses are a wide group of disorders characterized by an anomalous deposit of mucin in the skin. Discrete papular lichen myxoedematous (DPLM) is an uncommon subtype included in the primary cutaneous mucinoses. Contrary to secondary mucinoses, the deposit of mucin in this subtype is the main feature that determines its clinical appearance.

## Patient and observation

**Figure 1:**
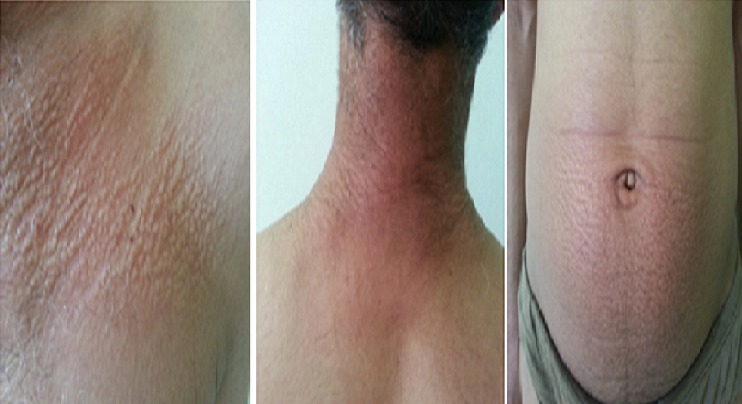
Multiple 2 – to 4 mm discrete, flesh – colored shiny papules without scales were present over the patient's left pectoral region,neck, and thehyopogastric

**Figure 2:**
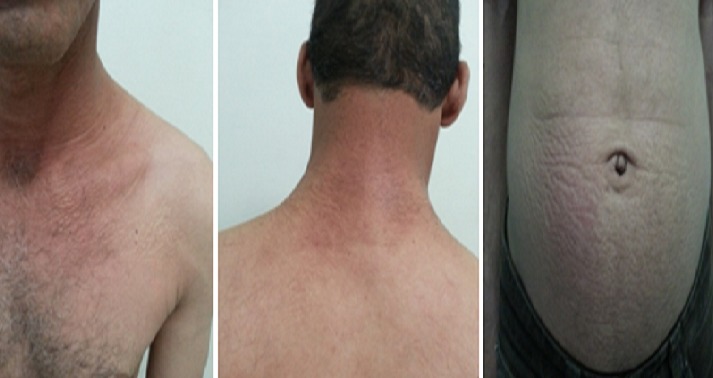
Good improvement after 4 weeks of topical corticosteroid

A 42-year-old man, without medical history, presented with slightly pruritic eruption of 3 month's duration. On medical examination, multiple 2 to 4 mm discrete, flesh – colored shiny papules without scales were present on the patient's bilateral posterior neck, left pectoral region and the hypogastric region (Figure 1). There were no other similar lesions on the rest of the body. The histologic examination showed a normal epidermis with deposit of mucin in the papillary and upper reticular dermis, sparing the deep dermis. There was an increased spacing between collagen bundles, but the number of fibroblasts was not increased. Laboratory investigations including blood count, liver enzymes, renal function, thyroid's hormones, serum protein analysis and immunoglobulins; were normal. Serology tests for human immunodeficiency virus(HIV), and hepatitis B and C viruses(HBV,HCV) were all negative. The patient was treated with topical corticosteroid with good improvement (Figure 2).

## Discussion

Lichen myxedematosus (LM), also called papular mucinosis, was first described by Dubreuilh in 1906 [1], and later classified by Montgomery and Underwood in 1953 [2]. In 2001, Rongioletti and Rebora [3] revised the classification system of LM, categorizing it into 3 broad subsets: (1) generalized papular and sclerodermoid, (2) localized LM, and (3) atypical forms. The first subset, generalized papular and sclerodermoid, represents scleromyxedema. Diagnosis requires a generalized papular and sclerodermoid eruption, monoclonal gammopathy (paraproteinemia), no evidence of thyroid dysfunction, and a histologictriad of fibroblast proliferation, fibrosis, and mucin deposition. Scleromyxedema is associated with many systemic disorders that may include numerous organ systems. Although spontaneous resolution has been reported, scleromyxedema typically is a long-term and disfiguring disease, associated with variable morbidity and mortality. The criteria for diagnosing the subset of localized LM requires a papular eruption, deposits of mucin with variable fibroblast growth, absence of paraproteinemia, and absence of thyroid dysfunction. There are 5 subtypes of the localized LM: discrete papular lichen myxedematosus (DPLM),acral persistent papular mucinosis (APPM),cutaneous mucinosis of infancy,self-healing papular mucinosis (SHPM), and nodular LM.

DPLM is a subtype of localized LM; it's a very rare entity, which affects both genders, however it has more effect on males than females [4]. There have been only 13 cases unrelated to HIV infection reported previously in the literature [5]. The aetiology of this disease remains unknown [3]. An over stimulation of fibroblasts has been implicated in patients with human immunodeficiency virus infection, but not in other cases [6]. DPLM is usually characterized by the presence of waxy, flesh-colored or reddish, violaceous papules, 2–5 mm in size, affecting the trunk and limbs in a symmetrical pattern. They can vary in number, and commonly appear in proximal sites, in contrast to persistent acral papular mucinosis, which appears at distal sites, and to scleromyxoedema, which shows generalized papular lesions [5]. Histologically, the upper and mid dermis shows edema and a diffuse or focal mucinous deposit under a normal epidermis. Fibroblast proliferation is variable, but there is neither collagen deposition nor sclerosis [3]. DPLM can be associated with HIV, Rongioletti et al [6] described 12 cases of DPLM in which HIV infection preceded the development of the skin lesions, or C hepatitis virus (HCV) especially among the Japanese population [7, 8]. DPLM never resolves on its own, but a transition to scleromyxoedema has not yet been reported [5]. It is difficult to treat a rare disease such as DPLMwhen the pathogenesis is unknown. Fortunately, DPLM and theother localized forms of LM are usually self-limitedto the skin and have very little or no morbidity, leading some experts to believe that the disorder is unnecessary to treat. Currently there is no effective treatment for DPLM andother types of localized cutaneous mucinoses. Many treatments have been tried including dermabrasion, CO2 laser, intralesional corticosteroids or hyaluronidase injections, oral retinoids and psoralen ultraviolet, A, pimecrolimus, with variable results [5]. Topical tacrolimus 0.1% has recently been suggested as an efficient alternative, given itscapability to inhibit both tumor necrosis factor-a andtransforming growth factor-b, thereby reducing the synthesis of glycosaminoglycans by fibroblasts [9].

## Conclusion

DPLM is a rare variant of localized LM. It is a self- limited skin disease, and prognosis is generally good. It is important for the clinicians to exclude any possible underlying disease such as gammopathies or HIV infection in such cases.
